# Molecular analysis of *TSC1* and *TSC2* genes and phenotypic correlations in Brazilian families with tuberous sclerosis

**DOI:** 10.1371/journal.pone.0185713

**Published:** 2017-10-02

**Authors:** Clévia Rosset, Filippo Vairo, Isabel Cristina Bandeira, Rudinei Luis Correia, Fernanda Veiga de Goes, Raquel Tavares Boy da Silva, Larissa Souza Mario Bueno, Mireille Caroline Silva de Miranda Gomes, Henrique de Campos Reis Galvão, João I. C. F. Neri, Maria Isabel Achatz, Cristina Brinckmann Oliveira Netto, Patricia Ashton-Prolla

**Affiliations:** 1 Laboratório de Medicina Genômica – Centro de Pesquisa Experimental – Hospital de Clínicas de Porto Alegre, Porto Alegre, Rio Grande do Sul, Brazil; 2 Programa de pós-graduação em genética e biologia molecular, Universidade Federal do Rio Grande do Sul, Porto Alegre, Rio Grande do Sul, Brazil; 3 Serviço de Genética Médica, Hospital de Clínicas de Porto Alegre, Porto Alegre, Rio Grande do Sul, Brazil; 4 Instituto Fernandes Figueira, Fundação Osvaldo Cruz, Rio de Janeiro, Rio de Janeiro, Brazil; 5 Hospital Universitário Pedro Ernesto, Universidade Estadual do Rio de Janeiro, Rio de Janeiro, Brazil; 6 Complexo Hospitalar Professor Edgard Santos, Salvador, Bahia, Brazil; 7 Hospital de Clínicas da Universidade de Campinas, Campinas, São Paulo, Brazil; 8 Hospital do Câncer de Barretos, Barretos, São Paulo, Brazil; 9 Centro Especializado em Reabilitação e Habilitação, Natal, Rio Grande do Norte, Brazil; 10 A.C. Camargo Cancer Center, São Paulo, São Paulo, Brazil; 11 Clinical Genetics Branch, Division of Cancer Epidemiology & Genetics, National Cancer Institute, National Institutes of Health, Rockville, United States of America; 12 Departamento de Genética - Universidade Federal do Rio Grande do Sul, Porto Alegre, Rio Grande do Sul, Brazil; University of Washington, UNITED STATES

## Abstract

Tuberous sclerosis complex (TSC) is an autosomal dominant multisystem disorder characterized by the development of multiple hamartomas in many organs and tissues. It occurs due to inactivating mutations in either of the two genes, *TSC1* and *TSC2*, following a second hit in a tumor suppressor gene in most hamartomas. Comprehensive screening for mutations in both the *TSC1* and *TSC2* loci has been performed in several cohorts of patients and a broad spectrum of pathogenic mutations have been described. In Brazil, there is no data regarding incidence and prevalence of tuberous sclerosis and mutations in *TSC1* and *TSC2*. We analyzed both genes in 53 patients with high suspicion of tuberous sclerosis using multiplex-ligation dependent probe amplification and a customized next generation sequencing panel. Confirmation of all variants was done by the Sanger method. We identified 50 distinct variants in 47 (89%) of the patients. Five were large rearrangements and 45 were point mutations. The symptoms presented by our series of patients were not different between male and female individuals, except for the more common occurrence of shagreen patch in women (p = 0.028). In our series, consistent with other studies, *TSC2* mutations were associated with a more severe phenotypic spectrum than *TSC1* mutations. This is the first study that sought to characterize the molecular spectrum of Brazilian individuals with tuberous sclerosis.

## Introduction

Tuberous sclerosis complex (TSC) (OMIM 191100) is an autosomal dominant multisystem disorder that occurs in all ethnic groups and both sexes. Population studies have estimated the prevalence of the disease in 1 in 6000 to 9000 individuals and at least 2 million people are affected worldwide [1]. The clinical findings and severity of TSC are highly variable; for this reason, clinical diagnostic criteria were established by a consortium in 1998 [2], and revised and updated by the same group in 2012 [3]. Most TSC patients have hamartomas in the brain, skin, kidneys, and heart. Involvement of the lung, gastrointestinal tract, bones, retina and/or gingiva is also common [4].

TSC occurs due to inactivating mutations in either of two genes, *TSC1* in chromosome 9q34 or *TSC2* in chromosome 16p13, and follows the two hit tumor suppressor model of pathogenesis in most hamartomas [5]. *TSC1* is composed of 23 exons and encodes for hamartin, a ubiquitously expressed 1164 amino acid protein [6] while *TSC2* consists of 42 exons and encodes for tuberin, a ubiquitously expressed 1807 amino acid protein [7]. Both proteins form a complex that regulates cell growth and tumorigenesis [8]. About one third of the patients with TSC have a familial form, in which the disorder follows a clearly dominant inheritance, whilst the other two-thirds are sporadic cases resulting from *de novo* germline mutations in one of the *TSC* genes [9, 10].

Comprehensive *TSC1* and *TSC2* mutation screening results have been reported in several cohorts of patients with TSC, as described in the Human Gene Mutation Database (HGMD) [11], and a broad spectrum of pathogenic mutations has been described. Among individuals who met the clinical criteria, 75–90% had an identifiable mutation in either *TSC1* or *TSC2*, and the majority of mutation-positive TSC patients have a mutation in *TSC2*. In sporadic cases, *TSC2* mutations are 2–10 times more common than *TSC1* mutations. In contrast, in multi-generation families segregating TSC, approximately half show linkage to each of the genes. About 80–95% of the *TSC1* and *TSC2* mutations are small mutations (missense, nonsense, small deletions, small insertions and splicing site mutations), and 5–20% are large duplications, large deletions or complex rearrangements. The high variability in mutation type and position renders molecular diagnosis of TSC challenging. This variability may explain, at least in part, the wide range of clinical symptoms observed in TSC patients, although timing and location of the second hit event is more likely to contribute to the variability of clinical symptoms. Several studies described possible genotype-phenotype correlations for TSC [10, 12–18]. Although, *TSC1*-related disease is usually less severe than *TSC2*-related disease.

In Brazil, there is no data regarding TSC incidence or *TSC1* and *TSC2* mutation prevalence amongst affected individuals. The genetic diagnosis is particularly important for patients with suspected TSC who do not fulfill clinical diagnostic criteria, and for genetic counseling. Therefore, the aims of this study were to describe demographics and clinical phenotype of patients with TSC from different Brazilian regions and characterize the germline *TSC1* and *TSC2* mutations observed in a group of individuals with clinical diagnosis of TSC.

## Methods

### Patients and DNA samples

Twenty-two male and 31 female individuals with clinically diagnosed or highly suspicion of TSC were recruited at eight Oncogenetics services from four different Brazilian regions, between August/2013 and May/2016. All patients were unrelated probands, including 17 familial and 36 sporadic cases. The study was approved by the institutional review board, Comitê de Ética em Pesquisa do Hospital de Clínicas de Porto Alegre (CEP-HCPA), under registration numbers GPPG 13–0260 and GPPG 15–0049. All individuals or legal representatives signed a written informed consent. Germline DNA samples were obtained from peripheral blood using a commercial kit (Flexigene Blood Kit, Qiagen, USA). Standardized clinical information was collected retrospectively by clinicians from each center after reviewing the medical records.

### Large deletion/duplication analysis

All 53 unrelated individuals were screened for large *TSC1* and *TSC2* deletions and duplications by SALSA Multiplex ligation-dependent probe amplification (MLPA) analysis. Commercial SALSA MLPA kits P124-C1 and P046-C1 (MRC-Holland, Amsterdam, The Netherlands) were used for *TSC1* and *TSC2* analysis, respectively, according to the manufacturer’s instructions. The P124-C1 and P046-C1 probe mixes contain probes for each of the *TSC1* and *TSC2* exons and 9 and 8 reference probes detecting different autosomal chromosomal locations, respectively. In addition, P046-C1 contains one probe for the *PKD1* gene, adjacent to *TSC2*, which is associated with polycystic kidney disease. DNA samples from healthy individuals were used as normal copy number controls. MLPA amplification products were separated on an ABI3500 capillary sequencer (Applied Biosystems, Foster City, CA, USA) and the results were analyzed using Coffalyser.net. Ratios <0.7 were considered deletions and ratios >1.4 were considered duplications. The chromosomal microarray technique CytoScan HD (Affymetrix, USA) was used to confirm MLPA analysis when a deletion/duplication larger than 300kb was identified by MLPA, as recommended by the manufacturer. The high-density, whole-genome CytoScan Array includes 2.69 million markers for copy number analysis. Chromosome Analysis Suite software (ChAS software 3.1) was used to analyze and visualize microarray data as well as for comparison of results with built-in reference of more than 400 samples.

### Next generation sequencing (NGS)

*TSC1* (NM_000368.4) and *TSC2* (NM_000548.3) amplicons were designed using the AmpliSeq Designer software (Thermo Fisher Scientific, CA, USA), targeting the complete coding sequence, 50 bp exon-intron junctions and 5' and 3' untranslated regions of *TSC1* and 99,83% of the coding sequence, 50 bp exon-intron junctions and 5' and 3' untranslated regions of *TSC2* gene, resulting in a total of 112 amplicons. A region of 17 base pairs of *TSC2* exon 29 remained uncovered. Amplicon library was prepared using the Ion AmpliSeq Library Kit 2.0 (Thermo Fisher Scientific, CA, USA) and NGS performed using 20ng of genomic DNA and an Ion 316 sequencing chip on an Ion Personal Genome Machine and the 200 Sequencing kit (Thermo Fisher Scientific, CA, USA), with 500 flows. Data from the Ion Torrent runs were analyzed using the platform-specific pipeline software Torrent Suite v3.2.1 for base calling, trim adapter and primer sequences and filtering out poor quality reads. The sequences were aligned to the hg19 human reference genome and for variant calling, the sequence runs were imported to the Ion Reporter software v5.0. Allele call frequency cutoff of 10% was used to investigate mosaic and non-mosaic germ-line variants. Phred score >500 was considered to filter variants. Variants were also reviewed and annotated using this software. Integrative genomics viewer was used for visualization of the mapped reads [19].

### Bioinformatics analysis

All variants identified by NGS were sought in the following databases: HGMD, ClinVar, Catalogue of Somatic Mutations in Cancer (COSMIC), Leiden Open Variation Database (LOVD), Tuberous Sclerosis Project database (TSP), and Genome Aggregation Database (gnomAD) [11, 20–24]. To predict the pathogenicity of missense variants and small indels we used two comprehensive *in silico* prediction tools: Mendelian Clinically Applicable Pathogenicity (M-CAP), and PredictSNP [25,26]. Splice site mutations were analyzed using BDGP software (Berkeley Drosophila Genome Project) and Mutation Taster [27, 28].

### NGS Validation by Sanger sequencing

For every sample with a variant of interest in one of the *TSC* genes, specific primers for the corresponding exon(s) were designed using Primer Blast (http://www.ncbi.nlm.nih.gov/tools/primer-blast/) and the reference sequences NM_000368.4 –*TSC1* and NM_000548.3 –*TSC2*. In addition, a specific primer for *TSC2* exon 29 (not covered in the NGS panel) was designed and DNA from all individuals was sequenced by the Sanger method for this exon. Primers were also designed for *TSC1* and *TSC2* promoter regions for variant screening in individuals with no identifiable pathogenic or probably pathogenic variants detected by MLPA or NGS. Primer sequences are available upon request. Forward and reverse primers were used to sequence the purified PCR products, using the BigDye Terminator v3.1 Cycle Sequencing Kit on an ABI 3500 Genetic Analyzer (Thermo Fisher Scientific, CA, USA). Sequences were aligned to their reference using CodonCode Aligner software implemented in MEGA 5.04. Variant calling and interpretation were based on the American College of Medical Genetics most recent guidelines [29]. Points were attributed to each variant according to these criteria, and they were classified as pathogenic, likely pathogenic, variant of uncertain significance (VUS), or likely benign.

### Statistical analysis

We compared the frequency of each clinical finding between male and female individuals. Statistical analysis was performed by conventional chi-squared or Fisher’s exact test using SPSS software (version 19.0).

## Results

We recruited 53 individuals with TSC from four different Brazilian regions (only the North region was not represented). Region of birth of the patients studied is summarized in S1A Fig, frequencies of the different types of mutation are in S1B Fig and individuals’ characteristics are shown in S1 Table. Median age at recruitment was 14 years (range: 6 months to 50 years) and average age at onset or recognition of the first symptoms was 3.3 years. Average age of TSC diagnosis was 7.1 years in familial cases and 2.6 years in sporadic cases. Fifty-two patients fulfilled the definitive TSC criteria established by the 1999 Tuberous Sclerosis Consensus Conference [2].

A phenotypic comparison between genders for each clinical feature is shown in Table 1. Only shagreen patch was more frequently observed in females (p = 0.028). This difference may occur due to random chance, since multiple comparisons were performed. Lymphangioleiomyomatosis (LAM) was only detected in one female patient. There were no differences in the frequency of any symptom when we compared familial and sporadic cases.

**Table 1:** Phenotypic comparison between male and female individuals with TSC.

	Male (n = 22)		Female (n = 31)		p-values
	P/N	%	P/N	%	
Median age (years) / interaquartile range	10 /17		15 /16		
Hypomelanotic macules	18/22	82	24/28	86	0.738
Facial angiofibromas	16/22	73	21/29	72	0.889
Confetti lesions	5/22	22	5/28	18	0.732
Shagreen patch	3/22	14	11/28	40	0.028*
Ungual fibromas	2/22	9	7/28	25	0.439
Renal angiomyolipoma	10/22	45	19/27	70	0.071
Multiple renal cysts	5/17	29	5/20	25	0.717
Cortical tubers	17/21	81	22/27	81	0.264
Seizures	12/15	80	10/15	67	0.682
Mental retardation	10/17	58	11/18	61	0.890
Subependymal nodules	7/21	33	15/26	58	0.161
Astrocytomas	3/21	14	7/26	27	0.731
Cardiac rhabdomyomas	7/21	33	9/27	33	0.927
Retinal hamartomas	3/20	15	2/27	7	0.638
Gingival fibromas	2/22	9	3/28	11	1.000
Dental pits	2/22	9	5/28	18	0.444
Hepatic angiomyolipoma	1/22	4	6/25	24	0.194
Lymphangiomyomatosis	0/22	0	1/28	5	0.246
Rectal polyp	0/20	0	2/28	8	0.504

**P** = presence (number of patients with the feature);

**N** = total number of patients examined;

**%** = frequency of each clinical feature in each group.

*Indicates statistically significant values.

Overall, 50 distinct variants were identified in 47 (89%) out of the 53 patients. MLPA analysis identified five (9%) patients who were heterozygous for large rearrangements in *TSC2*: four large deletions (7%) and one large duplication (2%). A complete *TSC2* deletion observed in one family was confirmed by CytoScan HD as a heterozygous deletion of 2.0Mb (108 genes including *TSC2* and *PKD1*). Two single exon deletions (exon 8 in *TSC1* and exon 19 in *TSC2*) were detected by MLPA. NGS and Sanger sequencing revealed point mutations at the hybridization probe sites of the specific exons, thus excluding the occurrence of these single-exon deletions. A *PKD1* deletion was found in one patient, since the MLPA kit P046 contains a probe for this gene. In addition, we identified 13 distinct heterozygous small variants in the coding region of *TSC1* and 32 in *TSC2*. We did not observe evidence of mosaicism (considering allele proportions between 10–50%). The read-depth achieved per amplicon per subject is shown in S2 Table. A summary of the pathogenic and likely pathogenic variants is in Table 2. Families with a likely benign or a VUS are shown in Table 3.

**Table 2:** Families with a pathogenic or likely pathogenic variant in *TSC1* and *TSC2*.

Family	Inheritance	Gene	Position	Coding change	Amino acid change	ClinVar/HGMD/LOVD/TSP (classification)	gnomAD MAF (homozygous)	M-CAP	Predict SNP1	ACMG*	Pathogenicity
1	Familial	*TSC1*	Exon 5	c.338T>A	p.(Leu113*)	No	NR	ND	ND	PVS1+PP1+PM2+PP4	Pathogenic
2	Familial	*TSC1*	Exon 8	c.682C>T	p.(Arg228*)	Yes (P)	NR	ND	ND	PVS1+PS4+PP1+PM2+PP4	Pathogenic
3	Sporadic	*TSC1*	Exon 8	c.733C>T	p.(Arg245*)	Yes (P)	NR	ND	ND	PVS1+PS4+PM2+PM6+PP4	Pathogenic
4	Sporadic	*TSC1*	Exon 9	c.801dup	p.(Glu268Argfs*5)	No	NR	ND	ND	PVS1+PM2+PM6+PP4	Pathogenic
5	Sporadic	*TSC1*	Exon 10	c.988del	p.(Leu330*)	No	NR	ND	ND	PVS1+PM2+PM6+PP4	Pathogenic
6	Familial	*TSC1*	Exon 10	c.989dupT	p.(Ser331Glufs*10)	Yes (P)	NR	ND	ND	PVS1+PS4+PP1+PM2+PP4	Pathogenic
7	Familial	*TSC1*	Intron 14	c.1439-2A>G	p.?	No	NR	ND	ND	PVS1+PP1+PM2+PP4	Pathogenic
8	Sporadic	*TSC1*	Exon 15	c.1888_1891del	p.(Lys630Glnfs*22)	Yes (P)	NR	ND	ND	PVS1+PS4+PM2+PM6+PP4	Pathogenic
9	Familial	*TSC1*	Exon 17	c.2071_2074dup	p.(Arg692Profs*15)	No	NR	ND	ND	PVS1+PP1+PM2+PP4	Pathogenic
10	Sporadic	*TSC1*	Exon 17	c.2090T>A	p.(Leu697*)	No	NR	ND	ND	PVS1+PM2+PM6+PP4	Pathogenic
11	Familial	*TSC1*	Exon 18	c.2287C>T	p.(Gln763*)	No	NR	ND	ND	PVS1+PP1+PM2+PP4	Pathogenic
12	Sporadic	*TSC2*	Exons 1–10	*TSC2*del e1-e10	p.?	Yes (P)	NR	ND	ND	PVS1+PS4+PM2+PM6+PP4	Pathogenic
13	Sporadic	*TSC2*	Exons 3–6	*TSC2*del e3-e6	p.?	No	NR	ND	ND	PVS1+PM2+PM6+PP4	Pathogenic
14	Sporadic	*TSC2*	Exon 3	c.169del	p.(Arg57Alafs*4)	No	NR	ND	ND	PVS1+PM2+PM6+PP4	Pathogenic
15	Familial	*TSC2*	Exon 8	c.724delinsTCCT	p.(Thr242delinsSerSer)	No	NR	ND	ND	PP1+PM2+PM4+PP4	Likely Pathogenic
16	Familial	*TSC2*	Exon 10	c.911G>A	p.(Trp304*)	Yes (NP)	NR	ND	ND	PVS1+PP1+PM2+PP4	Pathogenic
17	Sporadic	*TSC2*	Intron 10	c.975 + 1G>T	p.?	Yes (P)	NR	ND	ND	PVS1+PS4+PM2+PM6+PP4	Pathogenic
18	Sporadic	*TSC2*	Intron 10	c.976-15G>A	p.?	Yes (P)	NR	ND	ND	PS4+PM2+PM6+PP3+PP4	Pathogenic
19	Sporadic	*TSC2*	Exon 11	c.1008T>G	p.(Tyr336*)	Yes (NP)	NR	ND	ND	PVS1+PM2+PM6+PP4	Pathogenic
20	Familial	*TSC2*	Exon 11	c.1019T>C	p.(Leu340Pro)	Yes (P)	NR	P	D	PS4+PP1+PM2+PP3+PP4	Pathogenic
21	Sporadic	*TSC2*	Exon 12	c.1239_1240ins15	p.(Arg413_Cys414insValGlnPro*)	No	NR	ND	ND	PVS1+PM2+PM6+PP4	Pathogenic
22	Sporadic	*TSC2*	Exon 13	c.1323G>A	p.(Trp441*)	No	NR	ND	ND	PVS1+PM2+PM6+PP4	Pathogenic
23	Sporadic	*TSC2*	Exon 15	c.1513C>T	p.(Arg505*)	Yes (P)	NR	ND	ND	PVS1+PS4+PM2+PM6+PP4	Pathogenic
24	Familial	*TSC2*	Exon 16	c.1693del	p.(Leu565Trpfs*133)	Yes (NP)	NR	ND	ND	PVS1+PP1+PM2+PP4	Pathogenic
25	Sporadic	*TSC2*	Exons 17–33	*TSC2* dup e17-e33	p.?	No	NR	ND	ND	PVS1+PM2+PM6+PP4	Pathogenic
26	Familial	*TSC2*	Exon 19	c.1976_1977insA	p.(Ser660Glnfs*43)	No	NR	ND	ND	PVS1+PP1+PM2+PP4	Pathogenic
27	Familial	*TSC2*	Exon 19	c.2071del	p.(Arg691Alafs*7)	Yes (P)	NR	ND	ND	PVS1+PS4+PP1+PM2+PP4	Pathogenic
28	Sporadic	*TSC2*	Exon 20	c.2194C>T	p.(Gln732*)	Yes (P)	NR	ND	ND	PVS1+PS4+PM2+PM6+PP4	Pathogenic
29	Sporadic	*TSC2*	Intron 21	c.2355+1_2355+4del	p.?	Yes (P)	NR	ND	ND	PVS1+PS4+PM2+PM6+PP4	Pathogenic
30	Sporadic	*TSC2*	Exon 23	c.2551_2570dup	p.(Tyr857*)	No	NR	ND	ND	PVS1+PM2+PM6+PP4	Pathogenic
31	Sporadic	*TSC2*	Exon 27	c.2974C>T	p.(Gln992*)	Yes (P)	NR	ND	ND	PVS1+PS4+PM2+PM6+PP4	Pathogenic
32	Familial	*TSC2*	Exon 31	c.3685C>T	p.(Gln1229*)	Yes (P)	NR	ND	ND	PVS1+PS4+PP1+PM2+PP4	Pathogenic
33	Familial	*TSC2*	Exon 31	c.3772_3778del	p.(Ala1258Argfs*65)	No	NR	ND	ND	PVS1+PS4+PP1+PM2+PP4	Pathogenic
34	Sporadic	*TSC2*	Exon 34	c.4235_4236del	p.(Pro1412Argfs*3)	No	NR	ND	ND	PVS1+PS4+PM2+PM6+PP4	Pathogenic
35	Familial	*TSC2*	Exon 34	c.4375C>T	p.(Arg1459*)	Yes (P)	NR	ND	ND	PVS1+PS4+PP1+PM2+PP4	Pathogenic
36	Sporadic	*TSC2*	Exon 37	c.4685T>C	p.(Leu1562Pro)	Yes (NP)	NR	P	D	PM2+PM6+PP3+PP4	Likely Pathogenic
37	Familial	*TSC2*	Exon 40	5138_5149del	p.(Arg1713_Ala1716del)	Yes (LP)	NR	ND	ND	PP1+PM2+PM4+PP4+PP5	Pathogenic
38	Sporadic	*TSC2*	Exon 40	c.5106_5107insCACA	p.(Val1703Thrfs*4)	No	NR	ND	ND	PVS1+PM2+PM6+PP4	Pathogenic
39	Sporadic	*TSC2*	Exon 40	c.5126C>T	p.(Pro1709Leu)	Yes (P)	NR	P	D	PS4+PM2+PM6+PP3+PP4+BP1	Pathogenic
40	Sporadic	*TSC2/PKD1*	Exons 41-42/*PKD1*	*TSC2*del e41-e42/PKD1	p.?	Yes (P)	NR	ND	ND	PVS1+PS4+PM2+PM6+PP4	Pathogenic
41	Sporadic	*TSC2*	Exon 41	c.5238_5255del	p.(His1746_Arg1751del)	Yes (P)	NR	ND	ND	PS4+PP1+PM2+PM4+PP4	Pathogenic
42	Sporadic	*TSC2/PKD1*	*TSC2/PKD1*	*TSC2/PKD1*del	p.?	Yes (P)	NR	ND	ND	PVS1+PS4+PM2+PM6+PP4	Pathogenic

HGMD: Human Gene Mutation Database; LOVD: Leiden Open Variation Database; TSP: Tuberous Sclerosis Project; MAF: Minor allele frequency; B: Benign; LB: Likely benign; VUS: Variant of uncertain significance; LP: Likely pathogenic; P: Pathogenic; NP: Not provided; NR: Not reported; ND: Not determined; N: Neutral; D: Deleterious.

*ACMG 2015 pathogenicity criteria: PVS1: very strong; PS1-4: strong; PM1-6: moderate; PP1-5: supporting. ACMG 2015 benign criteria: BA1: stand-alone; BS1-4: strong; BP1-6: supporting.

**Table 3:** Families with a likely benign or variant of uncertain significance in *TSC1* and *TSC*.

Family	Inheritance	Gene	Position	Coding change	Amino acid change	ClinVar/HGMD/LOVD/TSP (classification)	gnomAD MAF (homozygous)	M-CAP	Predict SNP1	ACMG*	Pathogenicity
43	Sporadic	*TSC1*	Intron 7	c.664-10A>C	p.?	No	NR	ND	ND	PM2+PM6+PP4	VUS
44	Sporadic	*TSC1*	Exon 23	c.3387C>T	p. (=)	Yes (B; LB; VUS)	233/277142 (1)	ND	ND	PP4+BP4+BP6+BP7+BS1	Likely benign
45	Familial	*TSC2*	Intron 10	c.975+8G>A	p.?	Yes (LB)	13/262922 (0)	ND	ND	PP1+PP4+BP6	VUS
40	Sporadic	*TSC2*	Exon 19	c.2011G>T	p.(Gly671Cys)	No	NR	P	N	PM2+PM6+PP4+BP1	VUS
6, 46	Sporadic	*TSC2*	Exon 33	c.3986G>A	p.(Arg1329His)	Yes (B;LB)	1797/271710 (36)	ND	ND	PP4+BP1+BP4+BP6+BS1	Likely benign
37	Familial	*TSC2*	Exon 34	c.4397C>T	p.(Ser1466Leu)	Yes (LB; VUS)	4/266180 (0)	P	N	PP1+PP3+PP4+BP6	VUS
35	Familial	*TSC2*	Exon 35	c.4527_4529del	p.(Phe1510del)	Yes (B; LB)	1409/276714 (10)	ND	ND	PP1+PP4+BP4+BP6+BS1	Likely benign

HGMD: Human Gene Mutation Database; LOVD: Leiden Open Variation Database; TSP: Tuberous Sclerosis Project; MAF: Minor allele frequency; B: Benign; LB: Likely benign; VUS: Variant of uncertain significance; LP: Likely pathogenic; P: Pathogenic; NP: Not provided; NR: Not reported; ND: Not determined; N: Neutral; D: Deleterious.

*ACMG 2015 pathogenicity criteria: PVS1: very strong; PS1-4: strong; PM1-6: moderate; PP1-5: supporting. ACMG 2015 benign criteria: BA1: stand-alone; BS1-4: strong; BP1-6: supporting.

**Fig 1:**
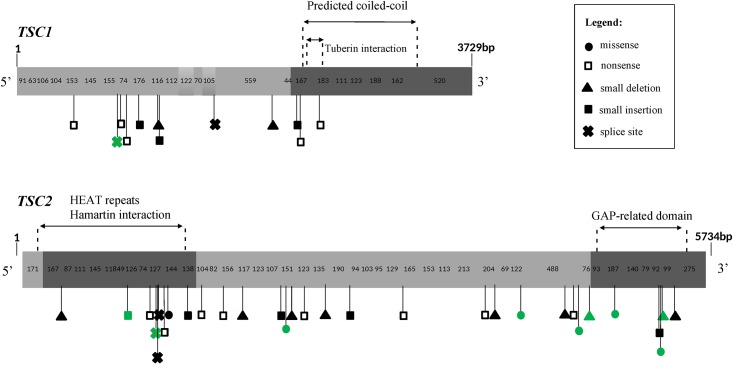
Distribution of *TSC1* and *TSC2* sequencing variants. The reference sequences used were NM_000368.4 for *TSC1* and NM_000548.4 for *TSC2*. The 3' unstranslated region of *TSC1* is not represented due to its large size. Synonymous variants are not shown. The alterations represented in green are variants of uncertain significance (VUS).

The distribution of small mutations within the *TSC1* and *TSC2* genes is shown in Fig 1. The tuberin domain that interacts with hamartin was recently solved and is shown accordingly [30]. We calculated the mean number of small mutations (including splice site changes) per nucleotide for each exon of both genes. The overall mutation frequency was higher at the *TSC2* locus (0.006 mutations per nucleotide) when compared to *TSC1* (0.003 mutations per nucleotide). Exons 11, 19, 34 and 40 of the *TSC2* gene had the highest frequency of mutations.

Considering clinical data, the most commonly observed skin/mucosal findings were hypopigmented macules, facial angiofibromas, shagreen patches and ungueal fibromas. Regarding central nervous system symptoms, the most common findings were cortical tubers, subependymal nodules, cognitive deficiency and seizures. Subependymal giant astrocytomas occurred in 23% of the patients. Other common findings were renal angiomyolipomas, multiple renal cysts and cardiac rhabdomyomas. We examined the clinical manifestations of patients with different types of mutations in different domains of the *TSC1* and *TSC2* to assess whether there was any correlation between mutation type and location in the gene with specific clinical features. Comparing the total number of individuals with a *TSC2* mutation and seizures with the number of individuals with *TSC1* mutation and seizures, individuals with *TSC2* mutation had a higher frequency of this symptom (p = 0.008). The same occurred when considering astrocytomas (p = 0.0038). We did not observe a difference in symptoms between patients with mutations in the first four exons of *TSC1* and in the region that codifies the coiled-coil domain. However, patients with nonsense variants, independently of the position in the gene, had cognitive impairment and seizures, while patients with other types of mutation do not have these symptoms. Regarding *TSC2*, the symptoms were similar in patients with mutations early in the protein, in the middle or in the GAP-related domain. The different types of mutations also did not result in specific phenotypes in this gene. For instance, the patient with an entire *TSC2* deletion had a similar phenotype to that of patients with point mutations.

Clinical data for patients with a synonymous variant or no mutation identified are summarized in S3 Table. All of these patients had at least two major diagnostic criteria for TSC and were sporadic cases. Cognitive impairment, subependymal giant cell astrocytomas, and retinal hamartomas did not occur in the group without an identifiable mutation. Other symptoms were also observed less frequently in this group, although the differences were not statistically significant.

## Discussion

This study sought to characterize the clinical and molecular profile of Brazilian individuals with tuberous sclerosis. Although many *TSC1* and *TSC2* disease-causing mutations have been identified in other populations, no studies including the Brazilian population have been undertaken. The overall mutation detection rate (89%) was within the expected in our study. Approximately two thirds of TSC probands worldwide are simplex cases [8]. Family history directly correlates with the presence of a deleterious mutation either in *TSC1* or *TSC2* [9, 31]. In our series, the majority of the probands (68%) had no family history of the disease (similar to other reports); of these cases, 63% had a variant in *TSC1* or *TSC2* identified. In the familial cases, 82% had an identifiable variant. In addition, distribution of mutations was also similar to other studies showing a predominance of rearrangements and point mutations in *TSC2* [32]. However, while *TSC2* mutations are 4–5 times more common than *TSC1* mutations in the literature, in our study *TSC2* mutations were only 2.5 times more common than *TSC1* mutations [9, 31]. The reason for the higher frequency of *TSC2* mutations in our population is currently unknown. The coding region of *TSC2* is about 50% larger than *TSC1*, the number of exons is nearly doubled, and the frequency of nonsense mutations and small indels are roughly proportional to difference in the gene size. In addition, *TSC2* has a much higher GC content than *TSC1* (60% vs. 43%), which could favor point mutation occurrence. On the other side, *TSC1* contains more repeat elements than *TSC2* (32% vs. 25% total sequence), which could favor the occurrence of gene rearrangements. However, *TSC2* rearrangements were seen in our cohort, while *TSC1* rearrangements were not. Mutations were distributed throughout all gene regions with the exception of the 3' region of *TSC2*. Fifteen variants occur in the hamartin or tuberin functional domains and all frameshift and nonsense alterations outside these domains create a stop codon that produces an incomplete protein with partial or no functional domains. There was a high occurrence of splice site mutations at the donor site of exon 10 of *TSC2*, and no higher frequency of other types of mutation in other gene regions.

Using a combined approach of NGS and rearrangement analysis by MLPA, we were able to identify 20 novel variants (8 in *TSC1* and 12 in *TSC2*) and 30 previously reported variants. Three deletions has already been described in the literature, including a large deletion including the *TSC2* and the *PKD1*. One possible explanation for the occurrence of a TSC phenotype with no identifiable germline *TSC1* or *TSC2* mutation in six probands (11%), could be related to intronic mutations distant from the exon-intron boundaries, which could affect the splicing process or gene regulation, causing a reduction of the normal mRNA transcript. In addition, somatic mosaicism could account for some of these cases, as described before [9], but this was not observed in any of the cases studied. However, we must emphasize that we reached a variant call frequency of >10%. Therefore, mosaics at a level of 10% or less variant call frequency would not have been detected, but it is not clear if mosaics at a level of 10% or less have clinical significance. Finally, a third genetic locus related to TSC could exist.

Results from several studies over the past few years have provided insights on how tuberin and hamartin might affect cell proliferation, growth, adhesion, migration, or protein trafficking. It has been demonstrated that tuberin and hamartin interact directly with each other, forming a cytoplasmic protein complex [33]. The C-terminal putative coiled-coil domain of hamartin is necessary for interaction with tuberin HEAT-repeat domain. Additionally, tuberin is phosphorylated at serine and tyrosine residues in response to growth factors, which affects the interaction between hamartin and tuberin [34]. The GAP-related domain (Fig 1) of tuberin is responsible for the inhibition of cell division by indirect modulation of mammalian target of rapamycin (mTOR), a central regulator of translation [35]. Considering the importance of these domains, mutations in the interaction domains or GAP-related domain, as well as in phosphorylated residues in tuberin or loss of function mutations that exclude these domains are likely to be pathogenic. Missense and splice site mutations may also affect directly these domains or interfere in protein folding, charge and hydrophobicity. Although we did not find mutations in tuberin phosphorylation sites, we identified mutations that affect hamartin or tuberin functional domains. Furthermore, all nonsense mutations observed cause a premature stop codon that excludes an important functional domain. Nonsense-mediated decay (NMD) could also explain the loss of function effect of nonsense and frameshift variants in *TSC1* and *TSC2*. Canonical splice site changes were classified as potentially pathogenic by bioinformatics algorithms and have functional tests already described in literature that proved their pathogenicity (they exclude the corresponding exons of the genes): c.975 + 1G>T and c.976-15G>A in *TSC2* and c.1439-2A>G in *TSC1* [36–38]. The mutation c.2355+1_2355+4del in *TSC2* that may also affect splice site has been reported as pathogenic after functional validation [39]. The other splice site variants found in this study are predicted by *in silico* tools to not change the splicing.

It is always difficult to predict the effect of missense variants on protein function. Analysis of familial segregation may help, but the progressively small size of families, lack of family history information, and the predominance of simplex cases make segregation analysis challenging. We chose to use two *in silico* prediction tools that combine several pathogenicity scores to achieve a consensus classification and try to reduce misclassification of the variants. M-CAP uses existing pathogenicity likelihood scores and direct measures of evolutionary conservation to achieve a misclassification rate of the pathogenic variants of less than 5%. PredictSNP^1^ is a consensus classifier that combines six tools and provides significant improvement in prediction performance over the individual tools and over other consensus classifiers, such as CONDEL and Meta-SNP [40, 41]. We were able to classify as pathogenic or likely pathogenic three of the six missense variants found in our probands: the *TSC2* exon 11 (c.1019T>C) variant has functional studies indicating significant lower *TSC2* expression [42]. The mutations p.Leu1562Pro and p.Pro1709Leu are localized in the GAP related domain of tuberin. Prolines are known to have a very rigid structure, sometimes forcing the backbone into a specific conformation. In the first mutation, a change from a leucine to a proline could disturb the GAP domain conformation; in the latter, the mutant residue is bigger and could lead to bumps in this functional domain. Additionally, a functional study showed that this mutation increases the ratio of T389-phosphorylated to total S6K when comparing to wild-type *TSC2*, which corresponds to an increase in mTORC1 activity [43]. The other three missense variants (p.Gly671Cys, p.Arg1329His and p.Ser1466Leu) are outside functional domains and occur concomitantly with other pathogenic mutations in *TSC2*. Both p.Arg1329His and p.Ser1466Leu variants have been described at low frequencies in gnomAD. VUS detected in the present study would be good candidates for functional studies which could help to establish their pathogenicity.

Clinical presentation did not differ between the genders, and signs and symptoms of TSC were most commonly observed in adults. Dermatological, central nervous system, and renal findings are described as the most common clinical features, observed in over 80% of the patients, while cardiac rhabdomyomas are present in 50%, and lymphangioleiomyomatosis in 40% of the female patients [44]. The frequencies of the most common symptoms in our cohort of patient were similar to those previously described, with exception of lymphangioleiomyomatosis, which we observed only in one female patient. We observed that *TSC2* variants were associated with a more severe phenotypic spectrum when compared to *TSC1* variants, which is consistent with other studies [9, 31]. Although previous studies have found similar results, all statistical findings in our study may occur due to random chance, since multiple comparisons were performed and the sample size was limited. Finally, a previous study has described two individuals with a *TSC2-PKD1* deletion with severe renal manifestations and skin alterations of TSC [45]. We did not observe more severe renal symptoms in patients with *PKD1* deletion. We were unable to establish any additional meaningful genotype-phenotype correlations in this series, what could be due to the extensive molecular heterogeneity observed in this first series of Brazilian patients with TSC. Several limitations must be considered when analyzing the results of our study: (i) patients were classified as sporadic cases when no relatives presented symptoms of TSC; (ii) failure in the recruitment of patients’ relatives to make a complete mutation segregation analysis. This is particularly difficult in sporadic cases, when relatives do not have symptoms of TSC and need to be submitted to genetic tests; (iii) clinical data collection was performed carefully, but some characteristics were not evaluated in all patients, as shown in Table 1. This occur when evaluations are requested but not performed by the patients, or when the data is not available in the medical records. However, these limitations probably did not interfere in variant classification and genotype-phenotype correlation assessment. In 23% of the patients no pathogenic or likely pathogenic *TSC1* or *TSC2* germline variant was identified. The molecular cause of the TSC phenotype of these patients remains elusive.

## Conclusion

Genetic testing is currently part of the TSC diagnostic criteria [3]. In individuals with suspected TSC, clinical diagnosis is complicated by a high degree of phenotypic variability and the potential for a late onset of certain features of the disease. Thus, genetic testing can play an important role in diagnostic confirmation, enabling genetic counseling to families, and providing additional understanding towards the etiology of the disorder. We designed a molecular diagnosis strategy for TSC that showed an overall variant detection rate of 89%; 69% of the patients had a pathogenic or likely pathogenic variant. No specific genotype-phenotype correlations were established in this specific cohort, but we confirmed findings described in other populations. Early genetic diagnosis of patients with TSC will become more important as better therapeutic interventions become available.

## Supporting information

S1 Fig Origin of TSC patients and mutation frequencies in *TSC1* and *TSC2*. **A**. Places of birth of the patients studied are represented as green spots in the Brazilian map. **B**. Overall *TSC1* and *TSC2* mutation frequencies (above) and *TSC1* and *TSC2* mutation frequencies according to the region of birth (below).
(PPTX)

S1 Table Characteristics of the TSC patients included in this study according to their birth regions in Brazil. (DOCX)

S2 Table Read depth of Ion Torrent analysis *per* amplicon of *TSC1* and *TSC2*. (DOCX)

S3 Table Clinical phenotypes of TSC patients with a synonymous or without *TSC1* or *TSC2* mutations. (DOCX)
