# Supplementary material for: Molecular analysis of TSC1 and TSC2 genes and phenotypic correlations in Brazilian families with tuberous sclerosis
Source: PLoS One. 2017 Oct 2;12(10):e0185713. doi: 10.1371/journal.pone.0185713 (PMC5624610; doi:10.1371/journal.pone.0185713)
Supplement: S1 Fig — A. Places of birth of the patients studied are represented as green spots in the Brazilian map. B. Overall TSC1 and TSC2 mutation frequencies (above) and TSC1 and TSC2 mutation frequencies according to the region of birth (below). (PPTX) [file pone.0185713.s001.pptx]

## Slide 1
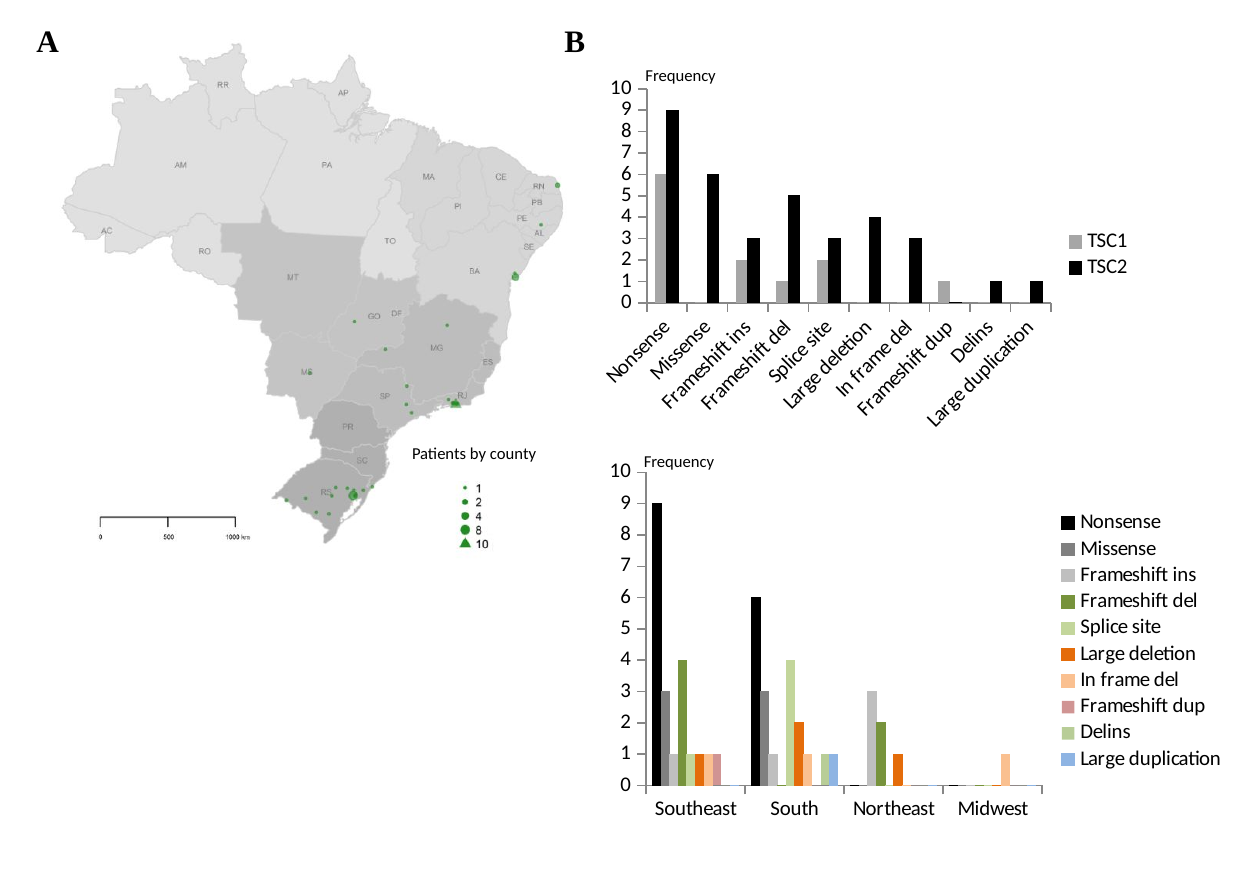

A
B
Frequency
### Chart
| Category | | |
|---|---|---|
| Nonsense | 6.0 | 9.0 |
| Missense | 0.0 | 6.0 |
| Frameshift ins | 2.0 | 3.0 |
| Frameshift del | 1.0 | 5.0 |
| Splice site | 2.0 | 3.0 |
| Large deletion | 0.0 | 4.0 |
| In frame del | 0.0 | 3.0 |
| Frameshift dup | 1.0 | 0.0 |
| Delins | 0.0 | 1.0 |
| Large duplication | 0.0 | 1.0 |Patients by county
Frequency
### Chart
| Category | Nonsense | Missense | Frameshift ins | Frameshift del | Splice site | Large deletion | In frame del | Frameshift dup | Delins | Large duplication |
|---|---|---|---|---|---|---|---|---|---|---|
| Southeast | 9.0 | 3.0 | 1.0 | 4.0 | 1.0 | 1.0 | 1.0 | 1.0 | 0.0 | 0.0 |
| South | 6.0 | 3.0 | 1.0 | 0.0 | 4.0 | 2.0 | 1.0 | 0.0 | 1.0 | 1.0 |
| Northeast | 0.0 | 0.0 | 3.0 | 2.0 | 0.0 | 1.0 | 0.0 | 0.0 | 0.0 | 0.0 |
| Midwest | 0.0 | 0.0 | 0.0 | 0.0 | 0.0 | 0.0 | 1.0 | 0.0 | 0.0 | 0.0 |
