# Supplementary material for: Molecular analysis of TSC1 and TSC2 genes and phenotypic correlations in Brazilian families with tuberous sclerosis
Source: PLoS One. 2017 Oct 2;12(10):e0185713. doi: 10.1371/journal.pone.0185713 (PMC5624610; doi:10.1371/journal.pone.0185713)
Supplement: S1 Table — (DOCX) [file pone.0185713.s002.docx]

**S1 Table.** Characteristics of the TSC patients included in this study according to their birth regions in Brazil.

| **Region** | **N** | **Median age (range)/ Interquartile range** | **Skin color**  **White/Black/Admixed/NI** | **Gender**  **M/F** | **Average age at onset (years)** | **Family history (Y/N)** |
| --- | --- | --- | --- | --- | --- | --- |
| Southeast | 22 | 15 (2-39) /14 | 15/0/4/3 | 10/12 | 2.0 | 8/14 |
| South | 21 | 14 (0-50) /27.5 | 18/1/2/0 | 11/10 | 6.6 | 6/15 |
| Northeast | 8 | 11.5 (1-25) /13 | 4/3/1/0 | 0/8 | 1.5 | 3/5 |
| Midwest | 2 | 19.5 (7-32) /25 | 2/0/0/0 | 1/1 | 29.0 | 0/2 |
| **Total** | **53** | **14 (0-50) /17.5** | **39/4/7/3** | **22/31** | **3.3** | **17/36** |

NI = Not informed.
