# Supplementary material for: Molecular analysis of TSC1 and TSC2 genes and phenotypic correlations in Brazilian families with tuberous sclerosis
Source: PLoS One. 2017 Oct 2;12(10):e0185713. doi: 10.1371/journal.pone.0185713 (PMC5624610; doi:10.1371/journal.pone.0185713)
Supplement: S2 Table — (DOCX) [file pone.0185713.s003.docx]

**S2 Table.** Read depth of Ion Torrent analysis *per* amplicon of *TSC1* and *TSC2*.

| **Family*** | ***TSC1*** | | |  | ***TSC2*** | | |
| --- | --- | --- | --- | --- | --- | --- | --- |
|  | **Average** | **Range** | **Median** |  | **Average** | **Range** | **Median** |
| 1 | 956 | 270-2099 | 938 |  | 841 | 35-2431 | 772 |
| 2 | 1223 | 365-2149 | 1224 |  | 1083 | 102-2607 | 1024 |
| 3 | 1864 | 605-4834 | 1786 |  | 1586 | 148-5258 | 1448 |
| 4 | 1321 | 441-2223 | 1306 |  | 1190 | 103-2134 | 1163 |
| 5 | 659 | 270-1243 | 637 |  | 548 | 53-1611 | 510 |
| 6 | 1827 | 428-4300 | 1753 |  | 1577 | 136-4249 | 1464 |
| 7 | 854 | 288-1290 | 871 |  | 577 | 55-1167 | 548 |
| 8 | 1747 | 355-2964 | 1711 |  | 1485 | 169-2626 | 1394 |
| 9 | 1464 | 289-9163 | 1144 |  | 1656 | 64-9703 | 1143 |
| 10 | 1368 | 370-3958 | 1286 |  | 1248 | 62-3994 | 1209 |
| 11 | 1018 | 267-1546 | 1050 |  | 841 | 110-1657 | 849 |
| 12 | 1875 | 516-2923 | 1896 |  | 1483 | 87-2379 | 1502 |
| 13 | 1480 | 300-3642 | 1423 |  | 1323 | 84-3405 | 1254 |
| 14 | 2270 | 633-4298 | 2129 |  | 1906 | 137-3244 | 1776 |
| 15 | 1242 | 440-2023 | 1225 |  | 1072 | 130-2073 | 1012 |
| 16 | 1177 | 409-4480 | 1093 |  | 1072 | 60-5488 | 889 |
| 17 | 1007 | 317-1497 | 1012 |  | 765 | 46-1414 | 746 |
| 18 | 1605 | 227-3537 | 1655 |  | 1672 | 140-3468 | 1630 |
| 19 | 1011 | 346-1828 | 1020 |  | 863 | 82-1814 | 827 |
| 20 | 1072 | 285-4767 | 927 |  | 1178 | 37-6780 | 812 |
| 21 | 840 | 201-5963 | 644 |  | 1094 | 55-8208 | 683 |
| 22 | 984 | 299-3712 | 900 |  | 968 | 48-5234 | 787 |
| 23 | 1057 | 323-2602 | 1046 |  | 955 | 73-2948 | 907 |
| 24 | 2517 | 772-4825 | 2594 |  | 2178 | 100-5157 | 2013 |
| 25 | 1395 | 407-4817 | 1266 |  | 1434 | 57-6345 | 1208 |
| 26 | 793 | 326-1809 | 767 |  | 691 | 44-2172 | 592 |
| 27 | 1814 | 356-3385 | 1836 |  | 1571 | 174-3276 | 1569 |
| 28 | 1801 | 224-3599 | 1839 |  | 1556 | 162-2690 | 1479 |
| 29 | 467 | 153-787 | 478 |  | 361 | 73-684 | 347 |
| 30 | 3596 | 1246-6390 | 3536 |  | 3094 | 157-6346 | 3073 |
| 31 | 875 | 275-1304 | 890 |  | 730 | 54-1399 | 709 |
| 32 | 1212 | 408-2079 | 1216 |  | 1006 | 59-2360 | 983 |
| 33 | 1059 | 458-3121 | 1023 |  | 941 | 78-3515 | 848 |
| 34 | 2266 | 716-7444 | 2145 |  | 2079 | 143-8432 | 1850 |
| 35 | 1091 | 355-1698 | 1102 |  | 870 | 49-1703 | 839 |
| 36 | 1362 | 521-2130 | 1382 |  | 541 | 75-1191 | 519 |
| 37 | 1179 | 271-2947 | 1186 |  | 966 | 44-3006 | 912 |
| 38 | 1175 | 409-1841 | 1169 |  | 909 | 133-1903 | 898 |
| 39 | 2045 | 423-8975 | 1773 |  | 2002 | 144-9632 | 1603 |
| 40 | 1869 | 372-4791 | 1776 |  | 1637 | 89-4044 | 1509 |
| 41 | 1530 | 297-4190 | 1445 |  | 1372 | 92-3917 | 1294 |

**S2 Table.** Cont.

| **Family*** | ***TSC1*** | | |  | ***TSC2*** | | |
| --- | --- | --- | --- | --- | --- | --- | --- |
|  | **Average** | **Range** | **Median** |  | **Average** | **Range** | **Median** |
| 42 | 1146 | 394-2237 | 1117 |  | 897 | 86-2481 | 877 |
| 43 | 1012 | 210-1788 | 1016 |  | 856 | 104-1704 | 813 |
| 44 | 1434 | 520-2204 | 1498 |  | 1156 | 122-2208 | 1118 |
| 45 | 1706 | 516-3927 | 1638 |  | 1578 | 93-3872 | 1513 |
| 46 | 1220 | 510-1889 | 1200 |  | 790 | 36-1734 | 753 |
| 47 | 3342 | 1022-12233 | 3181 |  | 3337 | 154-15628 | 2828 |
| 48 | 2355 | 767-6040 | 2236 |  | 2141 | 145-8027 | 1953 |
| 49 | 911 | 309-1472 | 885 |  | 709 | 41-1674 | 674 |
| 50 | 1496 | 457-2245 | 1528 |  | 1202 | 63-2443 | 1178 |
| 51 | 1103 | 261-5847 | 952 |  | 1179 | 54-7816 | 866 |
| 52 | 1218 | 406-1916 | 1228 |  | 891 | 113-1558 | 893 |
| 53 | 771 | 251-1206 | 810 |  | 588 | 57-1066 | 558 |

*Families 1-46 are the same indicated in Tables 3 and 4.
