# Supplementary material for: Molecular analysis of TSC1 and TSC2 genes and phenotypic correlations in Brazilian families with tuberous sclerosis
Source: PLoS One. 2017 Oct 2;12(10):e0185713. doi: 10.1371/journal.pone.0185713 (PMC5624610; doi:10.1371/journal.pone.0185713)
Supplement: S3 Table — (DOCX) [file pone.0185713.s004.docx]

**S3 Table.** Clinical phenotypes of TSC patients with a synonymous or without *TSC1* and *TSC2* mutations.

| **Inheritance** | **Gender** |  | **Brain** | | | | |  | **Skin** | | | |  | **Eyes** |  | **Kidneys** | |  | **Heart** |  | **Lungs** |  | **Liver** |
| --- | --- | --- | --- | --- | --- | --- | --- | --- | --- | --- | --- | --- | --- | --- | --- | --- | --- | --- | --- | --- | --- | --- | --- |
|  |  |  | **MR** | **SZ** | **CT** | **SEN** | **SEGA** |  | **FA** | **HM** | **SP** | **UF** |  | **RH** |  | **AL** | **MC** |  | **CR** |  | **LAM** |  | **HA** |
| Sporadic | M |  | **-** | **+** | **-** | **-** | **-** |  | **-** | **+** | **-** | **-** |  | **-** |  | **-** | **-** |  | **-** |  | **-** |  | **-** |
| Sporadic | M |  | **-** | **-** | **-** | **-** | **-** |  | **-** | **+** | **-** | **-** |  | **-** |  | **+** | **-** |  | **-** |  | **-** |  | **-** |
| Sporadic | M |  | **-** | **+** | **+** | **-** | **-** |  | **-** | **+** | **-** | **-** |  | **-** |  | **-** | **-** |  | **+** |  | **-** |  | **-** |
| Sporadic | F |  | **-** | **-** | **+** | **-** | **-** |  | **+** | **-** | **-** | **+** |  | **-** |  | **+** | **-** |  | **-** |  | **-** |  | **-** |
| Sporadic | F |  | **-** | **-** | **-** | **-** | **-** |  | **+** | **+** | **-** | **+** |  | **-** |  | **-** | **-** |  | **-** |  | **-** |  | **-** |
| Sporadic | F |  | **-** | **-** | **+** | **+** | **-** |  | **-** | **+** | **-** | **-** |  | **-** |  | **-** | **-** |  | **+** |  | **-** |  | **-** |
| Sporadic* | M |  | **-** | **+** | **+** | **-** | **-** |  | **+** | **-** | **-** | **-** |  | **-** |  | **+** | **+** |  | **-** |  | **-** |  | **-** |
| Sporadic | M |  | **-** | **-** | **-** | **-** | **-** |  | **+** | **+** | **-** | **-** |  | **-** |  | **+** | **-** |  | **-** |  | **-** |  | **-** |

*Patient with a *PKD1* gene deletion detected by MLPA. This is a single probe deletion not confirmed by other technique. **MR** = mental retardation; **SZ =** seizures; **CT =** cortical tuber(s); **SEN** = subependymal nodules; **SEGA** = subependymal giant cell astrocytoma; **FA** = facial angiofibromas; **HM** = hypopigmented macules of the skin; **SP** = shagreen patches; **UF** = ungual and periungual fibromas; **RH** = retinal hamartomas; **AL** = angiomyolipomas; **MC** = multiple cysts; **CR** = cardiac rhabdomyomas; **LAM** = lymphangiomyomatosis; **HA** = hepatic angiomyolipomas.
